# Intra-bone donor lymphocyte infusion at relapse: clinical outcome is associated with presence of CD8+ cells in the marrow

**DOI:** 10.1038/s41409-019-0632-z

**Published:** 2019-08-27

**Authors:** Andrzej Lange, Iwona Wodzińska-Maszko, Helena Pakos, Anna Sobczyńska-Konefał, Janusz Lange, Monika Mordak-Domagała, Jolanta Bocheńska, Emilia Jaskuła

**Affiliations:** 1 L. Hirszfeld Institute of Immunology and Experimental Therapy, Polish Academy of Sciences, Wroclaw, Poland; 2 Lower Silesian Center for Cellular Transplantation with National Bone Marrow Donor Registry, Wroclaw, Poland

**Keywords:** Leukaemia, Translational research

A recent EBMT paper documented the role of cellular therapy in fighting leukemia at relapse after alloHSCT [1]. In view of that, here, we present our study on the use of donor lymphocyte infusion (DLI) in relapsing patients after alloHSCT with a novel approach using the intra-bone (IB) route to diminish the risk of GvHD [2], and reinforce the pool of cytotoxic cells in the marrow, providing a direct contact between donor lymphocytes and blasts [3, 4].

Eighteen patients (all treated and observed in the Lower Silesian Center of Cellular Transplantation) relapsed in years Oct 2014–Nov 2018 and they were informed about the options in the treatment ahead. Nine patients decided to follow the standard way and left on chemotherapy at relapse (FLAM, FLAG, GMALL, Clofarbine+ AraC, Hydroxyurea supported by Sorafenib or Imatinib as appropriate) and nine for IB-DLI (Table 1).

**Table 1:** Patients’ characterics

Patient	Diagnosis	Donor (HSCT/ IB-DLI), HLA match	Relapse [months after HSCT], genetic status at relapse	Upfront therapy	Status at the beginning of IB-DLI/blast cells [%]	Number of IB-DLI infusions	IB-DLI dose of CD3+ cells/kg body weight	IB-DLI Intervals [days]	Volume [ml]	Outcome/cause of death	Survival from relapse [days]	General assessment of IB-DLI response
UPN 959, 22-years-old male	AML, 46XY, del. 7q,	SIB, 10/10	24 months, del 7q	IB-DLI	NR/26.31%	5	5.00E + 06	0	1	No CR reached, death/leukemia	421	Poor responder
							1.40E + 07	16	6			
							1.00E + 08	40	32 (to both crests)			
							6.94E + 07	227	12			
							3.64E + 06	95	1			
UPN 1006, 25-years-old male	AML, FLT3 -ITD + , 46 XY, KMT2A (MLL) aber., del. TP53	MUD, 9/10	9 months, FLT3-ITD + , 42~45,XY,add(8)(p1?),-?17[cp7]/46,XY(3)	FLAG	CR/3.56%	4	1.66E + 06	0	19	CR reached after 1^st^ until 4^th^ IB-DLI, death/leukemia	495	Good responder
							5.20E + 06	14	17			
							1.92E + 06	43	8			
							2.56E + 06	110	6			
UPN 909, 50-years-old female	AML, FLT3- ITD -, 46,XX	SIB, 10/10	42 months, FLT3-ITD -, 46,XX	IB-DLI alternately with 5′-azacitidine followed by 2nd HSCT	PR/11.98%	3	1.68E + 06	0	20	No CR reached, death/leukemia	243	Poor responder
							5.89E + 08	77	13			
							5.57E + 08	3	7			
UPN 725, 64-years-old male	CLL	SIB, 10/10	83 months, del TP53, del13q	IB-DLI	NR/50% of CD5 + CD19 + cells	5	1.00E + 06	0	6.5	No CR reached, death/toxicity of the 3^rd^ HSCT	865	Poor responder
							1.00E + 07	28	24			
							1.00E + 07	35	3			
							2.28E + 07	63	33			
							1.00E + 06	266	10			
UPN 1072, 25-years-old, male	AML, FLT3-ITD + , 46,XY,t(6;9)(q2?1;q34)	SIB, 10/10	6 months, FLT3-ITD +	FLAG	CR/0.75%	2	1.00E + 06	0	6.5	CR after at 1^st^ and 2^nd^ DLI, death/bronchiolitis obliterans	604	Good responder
							1.00E + 07	55	9			
UPN 1054, 38-years-old, female	AML, FLT3-ITD + , NPM1 +	MUD 10/10	9 months, extramedullary manifestation (skin) 16 months, FLT3-ITD + , 46,XX	skin relapse—local radiotherapy 20 Gy and D + A 2 + 5 regimen	Extra-medullary relapse Marrow CR/1.57%	3	4.68E + 05	0	10.5	CR reached after 2^nd^ DLI, alive	731	Good responder
							4.88E + 06	78	7			
							2.37E + 07	35	1.5			
UPN 1077, 58-years-old, female	CMML, MECOM aber.	MUD, 10/10	lack of response to alloHSCT	IB-DLI	NR/21%	1	5.00E + 05		1.25	No CR reached, death/leukemia	176	Poor responder
UPN 1050, 50-years-old, female	AML, FLT3-ITD -	SIB, 10/10	21 months, chimerism loss	IB-DLI	CR/0.67% (decreased chimerism)	1	9.98E + 04		9.5	CR, full chimerism after 1^st^ IB-DLI, alive	373	Good responder (chimerism back to 100%)
UPN 1074, 29-years-old, female	ALL	SIB, 10/10	11 months, extramedullary manifestation (kidney)	GMALL + radiotherapy–30 Gy for extramedullary relapse then IB-DLI	Extra-medullary relapse Marrow CR/0.34%	1	9.00E + 05		2.2	CR reached after 1^st^ IB-DLI, death/sepsis	239	IB-DLI good responder

*CR* complete remission, *PR* partial response, *NR* no response

Both groups were similar in age (range 22–64 vs. 28–59 years) and the proportion of AML/other hematologic malignancies (7/9 vs. 6/9), but differed in 12-months and 18-months survival after the relapse post alloHSCT which was favored by IB-DLI therapy (11% vs. 77% *p* = 0.006, and 11% vs. 55%, *p* = 0.035, respectively). The data presented here are in line with papers of Kharfan-Dabaja and El-Jurdi [5, 6]. This study in addition highlights the mechanism behind the positive effect of cellular therapy in patients relapsing post alloHSCT.

IB-DLI was usually started with a dose 1.0 × 10E6 CD3 (median) cells per kg of body weight and then in 1-month to 2-month intervals (median: 55 days) the infusions were repeated 2 to 5 times, escalating the dose in steps which a median value was 4.0 × 10E6 (Table 1). The cells for HSCT and DLI were obtained from the same donor being collected from the transplant material (stimulated) [7] or unstimulated if taken from the blood. DLI population (7-AAD− in 92% and CD3+ in 44%, median values) was injected into the posterior iliac crest under local anesthesia. The patients received low molecular weight heparin, i.v. paracetamol. Reported pain did not exceed 4 points on the 1–10 pain scale.

The blood and marrow work included cytometry and T cell receptor beta (TCR-beta) clonotype analysis using next generation sequencing, trephine bone marrow and skin biopsies were immunostained (see Supplementary file 1).

IB-DLI was given 2 to 5 times except in the case of a patient who failed to respond to the first DLI and the disease progress required chemotherapy and another one who responded favorably to IB-DLI which followed chemotherapy (GMALL B-ALL/NHL 2002 protocol) and did not require chemotherapy for 4 months but died due to sepsis. Altogether 25 infusions were performed. No unwanted effects were noted, none of the patients developed de novo GvHD. Two patients already had chronic GvHD. One patient with FLT3-ITD + AML received sorafenib when blasts reappeared during the IB-DLI treatment, which was withdrawn when blasts vanished. Another patient also having FLT3-ITD + AML received midostaurin as maintenance after a complete response to the chemotherapy and IB-DLI. In a CLL case after completion of 4 IB-DLI courses rituximab was implemented because of hemolytic anemia.

CD8+ cells (mean ± SEM: 36.49 ± 1.80% vs. 34.49 ± 1.90%, *p* = 0.002) as well as their PD-1+ (13.69 ± 1.25% vs. 10.67 ± 1.39%, *p* < 0.001) and CD69+ (10.08 ± 1.41% vs. 1.86 ± 0.22%, *p* < 0.001) subpopulations were higher in proportions in the marrow as compared to the blood.

In 14 trephine marrow biopsies taken from 5 patients with relapse after completion of 1–4 IB-DLIs revealed that CD8+ cells were spotted more frequently within the leukemic cell clusters than in the vicinity lacking leukemia counted in 5 high power fields (47 ± 7 vs. 18 ± 7 CD8+ cells, *p* = 0.005, Fig. 1). In the samples taken at the time of the response CD8+ cells were evenly distributed across the fields compared.

**Fig. 1:**
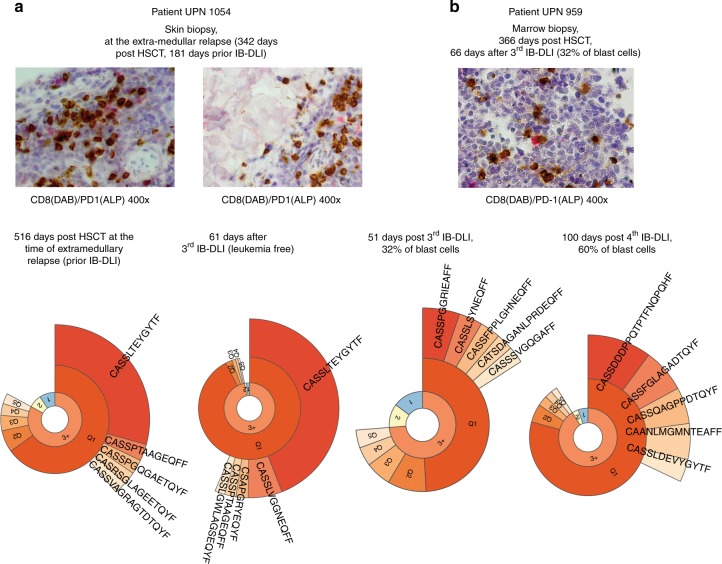
**a**. Infiltration of leukemic cells by CD8+ cells (brown) being partly PD-1 positive (red). The adjacent picture documents the presence of CD8+ cells within the leukemic cell infiltration but not in the vicinity lacking blasts. **b** CD8+ partly PD-1+ again within the leukemic cell cluster but in the patient who failed to respond. Below panel **a** the profiles of clonotypes are provided from the left; the first two represent a responding patient and show the growing prevalence of the immunodominant clones (top five); the next two pictures (**b**) represent a patient who failed; there is no repeatably found clone at lead in the 2 following observations but the diversity of the clonotypes is also shrunk (narrowing the yellow and blue segments, reflecting the presence of singletons and doubletons (first layer in the repertoire clonality plots). The top five TCR-beta clonotypes and their frequencies in the patients and the donors blood and marrow cells are given in the Supplementary file 2

A TCR-beta study was performed in the cells: (i) used for IB-DLI (7 samples), (ii) in the marrow samples prior to (4 samples) and after IB-DLI in 22 marrow samples and (iii) in 10 blood samples corresponding to the marrow within the same pair.

Different TCR-beta families were used by immunodominant clones (defined as the most frequent 5 clones) as compared within a pair of donor and recipient and even though the same TCR-beta V segment was used different pairing with J segment was employed. This translates into the profiles of immunodominant clones. Altogether 35 and 106 immunodominant clones were identified in donors and recipients, respectively, and only 3 of the same clonotypes were detected in two pairs. It shows that within a pair of donor and recipient different clones emerge in response to the local environment (Supplementary file 2). Among dominant clones 27 were identified as public clones [8] or those attributed to epitopes of viruses and reacting with MART-1 [9].

The blood and the marrow collected after IB-DLI were analyzed in the paired analysis for the TCR profile on 10 occasions. Analysis of the all clonotypes detected showed on the high degree of similarity between the marrow and the blood (Jensen-Shannon divergence (JSD) statistics: 0.004–0.037, median: 0.014). When the immunodominant clones were analyzed separately 32 clones were seen in the marrow as well as in the blood, 10 clones prevailed in the marrow and 22 in the blood.

The best response to the treatment was assessed when the AML patient reached at least a partial response—PR [10] lasting > = 4 months, in CLL if the lymphocyte count decreased ≥50% from baseline [11]. For poor responders the time point of comparison with the good responders was chosen as that with the lowest number of blasts after IB-DLI.

Five patients in the IB-DLI group responded well with remission lasting 240, 495, +373, 521, +731 days. Among these patients remission was supported either by one course of chemotherapy (1 patient) and/or by sorafenib or midostaurin given in 2 patients FLT3-ITD + with IB-DLI continuation. Finally, one patient died because of marrow relapse, while in two others extramedullary relapse of the modest progression appeared, but did not result in a fatal outcome. Two patients died due to nonmalignant conditions (sepsis, bronchiolitis obliterans).

Poor responders either responded shortly not reaching the PR criteria or reached PR which lasted less than 2 months.

Responders to IB-DLI were characterized by the following criteria, assessed before IB-DLI:

- a lower proportion of leukemic cells in the marrow as compared to poor responders (mean ± SEM: 1.61 ± 1.26% vs. 15.78 ± 2.21%, *p* = 0.016),
- higher numbers of CD8+ cells in the marrow (mean ± SEM:  3053 ± 1036 vs. 937 ± 47 × 10E6 cells/L (*p* < 0.070, post hoc ANOVA analysis)) and higher numbers of CD8 +  PD1+ cells (1238 ± 476 vs. 255 ± 73 × 10E6 cells/L, *p* < 0.06), as well as CD69 +  CD8+ cells (1154 ± 244 vs. 186 ± 63 × 10E6 cells/L, *p* < 0.015) than non-responding patients. A similar observation was reported by Bachireddy et al. [12, 13].

At the time of the best response in the responders group but not in those who failed the numbers in the marrow aspirates of CD8+ cells (median: 2401 vs. 1407 × 10E6 cells/L, *p* = 0.043) and their CD69+ (1290 vs. 189 × 10E6 cells/L, *p* = 0.043) and PD-1+ subpopulations (1134 vs. 230 × 10E6 cells/L, *p* = 0.043) cells decreased compared to the values prior to IB-DLI.

Figure 1 illustrates the cellular and genetic course seen in the patients receiving IB-DLI. CD8+ cells were PD-1 positive if the blasts emerge, these proportions decreased as the patients respond. When the TCR-beta clonotypes of the patients marrow lymphocytes prior to and at the best response time-point were compared there was no difference while comparing the whole pool (JSD varied from 0.014 to 0.150, median 0.034) but the difference appeared if only top 5 clonotypes were considered (some new clones preferentially expanded, JSD varied from 0.128 to 0.857, median: 0.186, *p* = 0.043).

The decrease in the proportion of blasts in the marrow is followed by lowering of the proportions of CD8+ cells including CD69+ cell subpopulation, as well as those of PD-1+. For maintaining the clones involved in the immune response the actual presence of target cells is needed so the decrease in the number of CD8+ cells and their PD-1+ subpopulation may be due to significant lowering in the proportions of blasts—target cells.

In 2 out of 9 patients receiving IB-DLI, GvHD manifesting in the skin was seen prior to implementation of this procedure including subacute GvHD in 1 case and sclerodermic like lesions in another one. The manifestation was not exacerbated by IB-DLIs. In the third case bronchiolitis obliterans was diagnosed, which appeared to be fatal 16 months after completion of the IB-DLI protocol.

IB-DLI was given as a first treatment attempt after relapse in 5 patients but 4 received chemotherapy at first (see Table 1). During the therapy tyrosine kinase inhibitors [14], 5′-azacitidine or anti-CD20 MoAb (CLL case) were given to optimize the treatment results. Therefore IB-DLI was used to facilitate the response and minimize the need for chemotherapy in heavily pre-treated patients. This approach was also favored by Vanneman [15], who showed that the parallel use of immune and targeted therapy may bring the best treatment effect.

The study was granted by the Bioethics Committee at the Medical University of Wroclaw (KB-369/2014) approval and supported by INNOMED/I/1/NCBR/2014, the patients signed an informed written consent.

## Supplementary information

Supplementary file 1

Supplementary file 2
