# Supplementary material for: Intra-bone donor lymphocyte infusion at relapse: clinical outcome is associated with presence of CD8+ cells in the marrow
Source: Bone Marrow Transplant. 2019 Aug 27;55(5):974–8. doi: 10.1038/s41409-019-0632-z (PMC7198473; doi:10.1038/s41409-019-0632-z)
Supplement: Supplementary file 1 [file 41409_2019_632_MOESM1_ESM.docx]

Methods:

The blood and marrow samples were taken prior to each IB-DLI for cytology, cytometry (MoAb for CD3, CD8, CD4, CD20, CD14, DR (BD, Erembodegen, Belgium) CD69 - clone FN50, CD279 - clone NIH4 (DB Pharmingen) and genetic work (chimerism, mutations, sequences of TCR-beta V(D)J variable regions). Trephine bone marrow and skin biopsies were stained with MoAb to CD3, CD8, CD4, CD14, CD19, CD117 (BD, Erembodegen, Belgium), and PD-1 positivity using EP 239 MoAb (Cell Marque, Rocklin, CA, USA).

TCR-beta genes’ expression was analyzed in a mononuclear cell population (Lymphoprep: d=1.077 g/mL; Nycomed Pharma AS, Oslo). RNA isolated using the TRI Reagent (Sigma-Aldrich, St. Louis, MO, USA) was sequenced using a SMARTer Human TCR a/b Profiling Kit (Takara Bio, Mountain View, CA, USA) in MiSeq (Illumina, San Diego, CA, USA). MiXCR (1) Immune Repertoire Analyzer (Illumina BaseSpace platform) and VDJtools (2) were used for analysis.

References:

1. Bolotin DA, Poslavsky S, Mitrophanov I, Shugay M, Mamedov IZ, Putintseva E V, et al. MiXCR: software for comprehensive adaptive immunity profiling. Nat Methods. 2015 May 1;12(5):380–1.

2. Shugay M, Bagaev D V., Turchaninova MA, Bolotin DA, Britanova O V., Putintseva E V., et al. VDJtools: Unifying Post-analysis of T Cell Receptor Repertoires. Gardner PP, editor. PLOS Comput Biol. 2015 Nov 25;11(11):e1004503.
